# A generalizable deep learning regression model for automated glaucoma screening from fundus images

**DOI:** 10.1038/s41746-023-00857-0

**Published:** 2023-06-13

**Authors:** Ruben Hemelings, Bart Elen, Alexander K. Schuster, Matthew B. Blaschko, João Barbosa-Breda, Pekko Hujanen, Annika Junglas, Stefan Nickels, Andrew White, Norbert Pfeiffer, Paul Mitchell, Patrick De Boever, Anja Tuulonen, Ingeborg Stalmans

**Affiliations:** 1 Research Group Ophthalmology, Department of Neurosciences, KU Leuven, Herestraat 49, 3000 Leuven, Belgium; 2 Flemish Institute for Technological Research (VITO), Boeretang 200, 2400 Mol, Belgium; 3 Department of Ophthalmology, University Medical Center Mainz, Langenbeckstr. 1, 55131 Mainz, Germany; 4 ESAT-PSI, KU Leuven, Kasteelpark Arenberg 10, 3001 Leuven, Belgium; 5 Cardiovascular R&D Center, Faculty of Medicine of the University of Porto, Alameda Prof. Hernâni Monteiro, 4200-319 Porto, Portugal; 6 Department of Ophthalmology, Centro Hospitalar e Universitário São João, Alameda Prof. Hernâni Monteiro, 4200-319 Porto, Portugal; 7 Tays Eye Centre, Tampere University Hospital, Tampere, Finland; 8 Department of Ophthalmology, The University of Sydney, Sydney, NSW Australia; 9 Centre for Environmental Sciences, Hasselt University, Agoralaan building D, 3590 Diepenbeek, Belgium; 10 University of Antwerp, Department of Biology, 2610 Wilrijk, Belgium; 11 Ophthalmology Department, UZ Leuven, Herestraat 49, 3000 Leuven, Belgium

**Keywords:** Optic nerve diseases, Translational research, Medical imaging, Diagnosis

## Abstract

A plethora of classification models for the detection of glaucoma from fundus images have been proposed in recent years. Often trained with data from a single glaucoma clinic, they report impressive performance on internal test sets, but tend to struggle in generalizing to external sets. This performance drop can be attributed to data shifts in glaucoma prevalence, fundus camera, and the definition of glaucoma ground truth. In this study, we confirm that a previously described regression network for glaucoma referral (G-RISK) obtains excellent results in a variety of challenging settings. Thirteen different data sources of labeled fundus images were utilized. The data sources include two large population cohorts (Australian Blue Mountains Eye Study, BMES and German Gutenberg Health Study, GHS) and 11 publicly available datasets (AIROGS, ORIGA, REFUGE1, LAG, ODIR, REFUGE2, GAMMA, RIM-ONEr3, RIM-ONE DL, ACRIMA, PAPILA). To minimize data shifts in input data, a standardized image processing strategy was developed to obtain 30° disc-centered images from the original data. A total of 149,455 images were included for model testing. Area under the receiver operating characteristic curve (AUC) for BMES and GHS population cohorts were at 0.976 [95% CI: 0.967–0.986] and 0.984 [95% CI: 0.980–0.991] on participant level, respectively. At a fixed specificity of 95%, sensitivities were at 87.3% and 90.3%, respectively, surpassing the minimum criteria of 85% sensitivity recommended by Prevent Blindness America. AUC values on the eleven publicly available data sets ranged from 0.854 to 0.988. These results confirm the excellent generalizability of a glaucoma risk regression model trained with homogeneous data from a single tertiary referral center. Further validation using prospective cohort studies is warranted.

## Introduction

Glaucoma is a leading cause of irreversible vision impairment, and it will further increase due to an ageing global population^1^. This growth will only add to the current high rate of over 50% of undetected cases in developed and developing countries^2–5^.

Current primary open-angle glaucoma (POAG) screening methods are not cost-effective in population-based settings, as they would generate a large number of false positives with a disease prevalence at 3.5% in populations aged 40–80 years^6–8^. This would overburden the health system, which is currently running at or above its capacity. Diagnosis is currently done opportunistically whenever a patient is seen by an eye health practitioner. This scenario cannot improve current rates of undiagnosed patients and, at the same time, identify those at higher risk of blindness. Screening solutions in the form of intra-ocular pressure (IOP) measurements miss glaucoma cases with normal tension, which can represent a high proportion of POAG^7,9,10^. Meanwhile, visual field testing is lengthy and produces highly variable results^11^. Glaucoma referral based on artificial intelligence (AI) analysis of digital fundus images has been proposed as a potential solution, given the modality’s widespread availability, low associated cost and non-invasive characteristic^12^. Moreover, convolutional neural networks (CNNs) can extract glaucomatous information from fundus images that exceed the capabilities of most human experts, such as the quantitative estimation of retinal nerve fiber layer thickness (RNFL)^13^ or glaucoma detection when the optic disc is removed from the image^14^.

AI-based glaucoma detection has been reported with high performance on internal validation, but the performance degraded in external testing conditions and, more specifically, in real-world settings^15–17^. Effective AI models trained on labeled fundus images from a single medical center need to be robust to distribution changes when deployed in new settings that feature Out-of-Distribution (OoD) data^18^. This requirement transcends the classical assumption in machine learning that train and test data come from the same distribution^19^. Such a data shift can arise when the model was trained on images captured with a particular fundus camera and tested on images from a second device. This inter-center heterogeneity in fundus images can be due to varying fields of view (FOV), color distribution, illumination, and area of interest (disc-centered or macula-centered). Differences in population, such as ethnicity, myopia prevalence and glaucoma prevalence are other common causes of data shifts that lead to degraded performance. Furthermore, a wide variety of glaucoma definitions exist, exacerbating the challenges related to OoD data. Solutions to counter data shifts, such as domain adaptation, have been described in the context of retinal image analysis, leading to improved generalizability^20,21^. However, these approaches often rely on the availability of labeled images from the target set during model development. This is typically not encountered in real-world applications, as these models should work on prospective data from new sources.

**Fig. 1:**
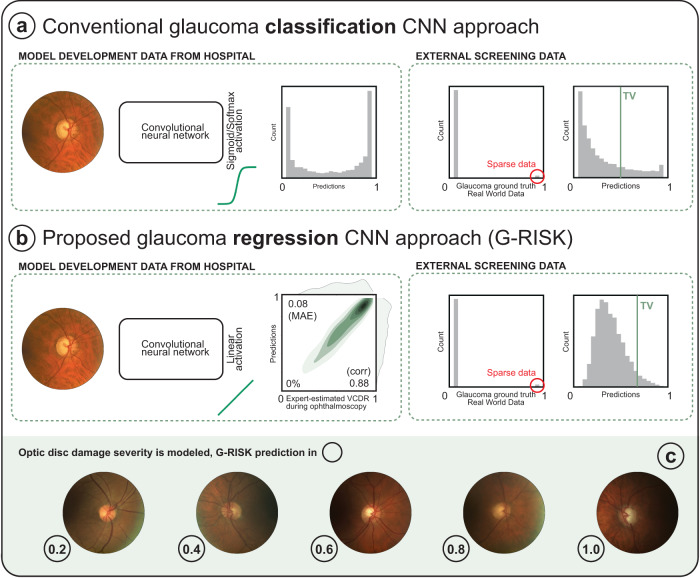
Overview of the G-RISK regression approach versus conventional glaucoma detection CNNs that are trained with binary labels. Both models were described in our previous work on explainable AI for glaucoma detection. The mismatch between the prevalence in a tertiary referral center (used for model development) and sparse real world data (external testing) leads to overprediction in the latter. The prediction histogram illustrates this phenomenon in the binary classification approach (**a**), with significantly more cases referred to as being glaucomatous than with G-RISK (**b**). Also note the spike in cases with prediction close to 1, versus a consistent decrease in cases as the prediction value increases for G-RISK. TV refers to the optimal threshold value. TV is typically fixed at 0.5 in binary classification models due to a sharp sigmoid/softmax activation function. In a regression approach with linear activation, TV can be set at a different value, depending on the costs associated with FP and FN. **c** Examples of fundus images with an increasing G-RISK score.

This work expands on the validation of convolutional neural networks (CNNs) for glaucoma screening from fundus images (Fig. 1). Instead of a CNN that performs binary classification (glaucoma or not), we opted for a regression CNN that outputs a continuous risk score. This risk score for CNN training was expert-estimated vertical cup-disc ratio (VCDR), which increases alongside glaucoma severity. The estimation of other glaucoma-related continuous biomarkers using regression CNNs has been described in related work, such as average RNFL thickness^13^ and Bruch’s membrane opening-minimum rim width (BMO-MRW)^22^. However, the analysis of thresholding those CNN-estimated variables against a glaucoma ground truth is limited.

The generalizability and robustness of our previously described glaucoma risk regression model (G-RISK)^14^ was assessed on fundus images from two large population cohorts, the Blue Mountains Eye Study (BMES)^2^ and the Gutenberg Health Study (GHS)^23^, as well as on eleven external publicly available data sets. The performance of the model was evaluated using the glaucoma ground truth defined by the data set owners, which varied widely, providing a comprehensive assessment of the model’s ability to adapt to different populations, imaging conditions, and ground truth definitions.

## Results

### Final data selection

**Fig. 2:**
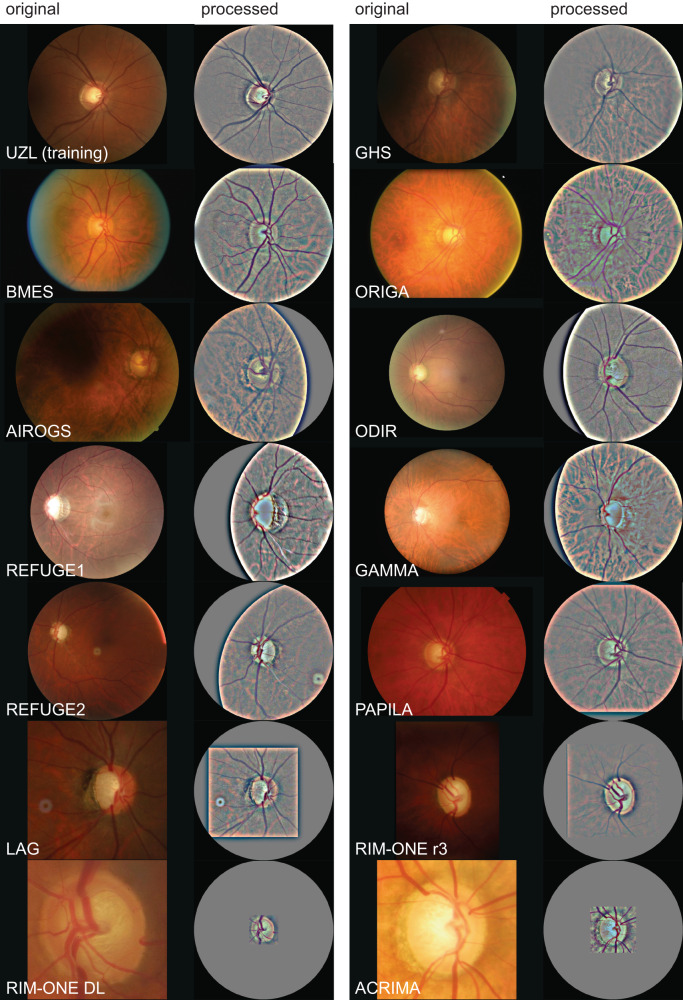
Examples of the training set and thirteen data sets used for external testing of G-RISK for generalizable glaucoma detection. Each pair displays a randomly selected original unprocessed image that features glaucoma-induced damage (left) and the corresponding 30° disc-centered result after image manipulation (right), prepared for G-RISK input.

G-RISK was validated on thirteen independent data sets from six countries, including three large screening cohorts. From the initial 151,145 color fundus images pool, a total number of 149,455 test images were included after quality control (removal rate of 1.12%, see “Methods”—*Image quality control*). Examples of image preprocessing are in Fig. 2, one before-after pair per data source. Glaucoma prevalence ranged from 1.08% in GHS to 56.17% in ACRIMA data.

### Population-based data

In the two population-based studies (BMES and GHS), the trained G-RISK obtained AUC scores of 0.976 [95% CI: 0.967–0.986] and 0.984 [95% CI: 0.978–0.986] on participant level, respectively. Harmonized sensitivity and specificity were at ~92.2% for BMES and ~94.2% for GHS when thresholding at 0.70 in both sets. For BMES, the AUC value was equal to 0.967 [95% CI: 0.956–0.979] on eye level. When maintaining a 95% specificity on participant level, sensitivity levels reached 87.3% and 90.3% on BMES and GHS, respectively.

### Publicly available data

The performance of the CNN model remained high in the publicly available data sets although being characterized by considerable heterogeneity in image capturing and glaucoma ground truth procedures. The lowest AUC value of 0.854 [95% CI: 0.821–0.886] was recorded on the complete ORIGA data (650 images), with balanced specificity and sensitivity at 78%. On the other side of the spectrum, the evaluation on GAMMA resulted in an AUC of 0.987 [95% CI: 0.971–1]. The CNN maintained performance (AUC of 0.917 [95% CI: 0.900–0.933]) in the challenging ODIR data set, which features additional ocular diseases, including diabetic retinopathy and age-related macular degeneration. Glaucoma prevalence in this set also approaches real-world distributions (4.70%). Detailed results for all data sources and subsets are in Table 1.

**Table 1:** Performance of G-RISK in external data sets.

Data	#cases	Prevalence (%)	AUC [95% CI]	Specificity	Sensitivity	Threshold value (TV)
Population-based studies						
BMES (eye)	6927	2.14	0.967 [0.956–0.979]	0.907 0.900 0.950 0.975	0.899 0.912 0.824 0.736	0.65 0.64 0.70 0.75
BMES (participant)	3554	2.87	0.976 [0.967–0.986]	0.923 0.900 0.950 0.975	0.922 0.922 0.873 0.765	0.70 0.67 0.73 0.77
GHS (participant)	11,528 (23,318 images)	1.08	0.984 [0.978–0.990]	0.938 0.900 0.950 0.975	0.935 0.984 0.903 0.806	0.70 0.67 0.72 0.75
Publicly available data set (image level, unless otherwise stated)						
AIROGS	100,022	3.11	0.963 [0.960–0.965]	0.900 0.900 0.950 0.975	0.899 0.899 0.798 0.675	0.74 0.74 0.78 0.82
ORIGA	650	25.85	0.854 [0.821–0.886]	0.778	0.774	0.66
REFUGE1 (all)	1200	10	0.952 [0.925–0.979]	0.871	0.892	0.58
(test set only)	400	10	0.986 [0.974–0.999]	0.963	0.925	0.61
ODIR	6193	4.70	0.917 [0.900–0.933]	0.845	0.842	0.75
REFUGE2 (val)	400	10	0.914	NA	NA	NA
REFUGE2 (test)	400	10	0.867	NA	NA	NA
GAMMA (all)	100	50	0.987 [0.973–1.000]	0.940	0.920	0.64
(early)	76	34.21	0.986 [0.969–1.000]	0.940	0.885	0.64
(advanced)	74	32.43	0.988 [0.969–1.000]	0.960	0.917	0.67
RIM-ONE r3 (w/o suspects)	124	31.45	0.973 [0.942–1.000]	0.882	0.897	0.66
(with suspects)	159	24.53	0.934 [0.889–0.978]	0.850	0.846	0.71
RIM-ONE DL (all)	485	35.46	0.972 [0.957–0.986]	0.914	0.913	0.66
(test set only)	174	32.18	0.952 [0.924–0.980]	0.881	0.857	0.70
PAPILA (eye) (susp. referable)	488	31.76	0.769 [0.722–0.815]	0.694	0.690	0.64
(susp. non-referable)	488	17.83	0.882 [0.840–0.923]	0.803	0.782	0.70
PAPILA (participant) (susp. referable)	244	33.20	0.813 [0.753–0.874]	0.767	0.728	0.71
(susp. non-referable)	244	19.26	0.924 [0.878–0.969]	0.878	0.851	0.78
ACRIMA*	705	56.17	0.884 [0.860–0.908]	0.793	0.790	0.64
LAG*	4854	35.25	0.926 [0.918–0.934]	0.853	0.853	0.62

### Prediction threshold analysis

Optimal threshold values (TV) for the publicly available data sets ranged from 0.58 (REFUGE1) to 0.75 (ODIR) on image level. TV was more elevated in sets that contained other pathologies (0.75 in the multi-disease set of ODIR and 0.74 in the diabetes population of AIROGS), or when glaucoma suspects were regarded as non-glaucomatous (0.66 to 0.71 in RIM-ONE r3, 0.64 to 0.70 in PAPILA). TV also increased when screening for advanced glaucoma compared to early glaucoma (0.67 versus 0.64 in GAMMA subsets). Finally, TV increased from image level (0.65) to participant level (0.70) on BMES data due to taking the maximum prediction of both eyes per individual. TV per data set can be retrieved from the last column of Table 1.

Using a fixed threshold value of 0.7 across all data sets does not impact the AUC score, but alters the sensitivity and specificity values. Table 2 shows specificity values range from 0.70 on PAPILA to 0.99 on REFUGE1 data. Sensitivity values fall between 0.68 (ORIGA) and 0.94 (GHS, PAPILA, AIROGS).

**Table 2:** Specificity and sensitivity values per data set when TV is fixed at 0.70.

Data	Specificity [95% CI]	Sensitivity [95% CI]
BMES (eye)	0.95 [0.95–0.96]	0.81 [0.74–0.87]
BMES (individual)	0.93 [0.92–0.93]	0.89 [0.82–0.94]
GHS (individual)	0.94 [0.93–0.94]	0.94 [0.89–0.97]
PAPILA (individual)	0.70 [0.63–0.76]	0.94 [0.83–0.98]
AIROGS	0.85 [0.85–0.85]	0.94 [0.93–0.95]
ORIGA	0.86 [0.83–0.89]	0.68 [0.61–0.75]
REFUGE1	0.99 [0.98–0.99]	0.77 [0.68–0.83]
ODIR	0.74 [0.73–0.75]	0.89 [0.85–0.92]
GAMMA	0.98 [0.90–1.00]	0.90 [0.79–0.96]
LAG	0.95 [0.94–0.95]	0.74 [0.72–0.76]
RIM-ONE r3	0.83 [0.76–0.89]	0.87 [0.73–0.94]
RIM-ONE DL	0.96 [0.93–0.97]	0.85 [0.79–0.89]
ACRIMA	0.87 [0.83–0.90]	0.73 [0.69–0.77]

Evaluation was done on image (eye) level unless otherwise stated.

**Fig. 3:**
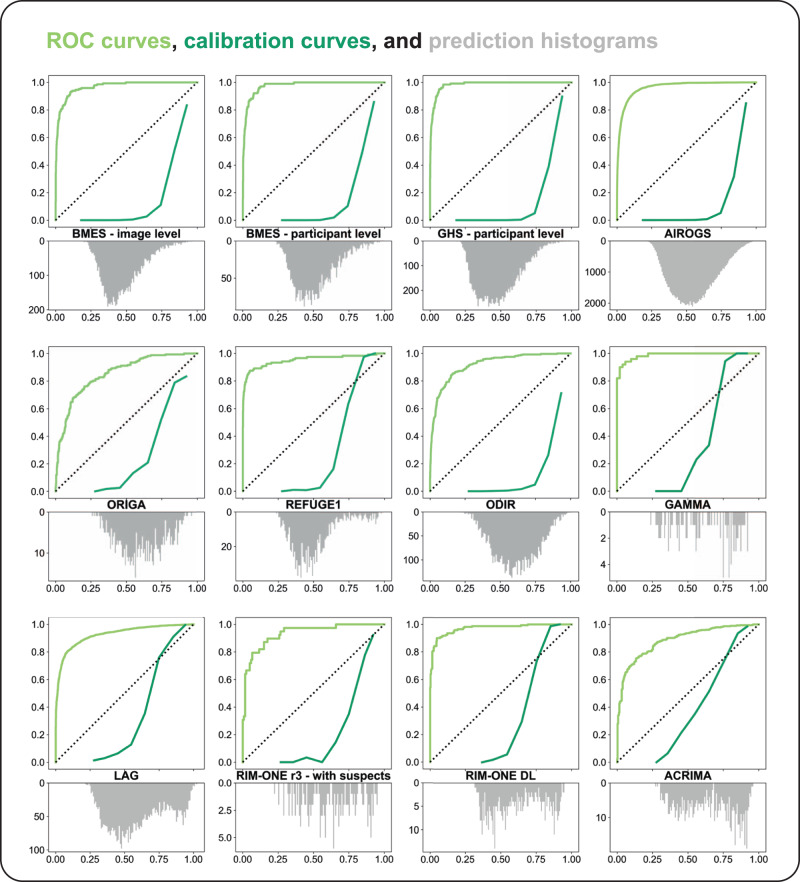
Combined ROC curve, calibration curve, and prediction histogram plots per data set that featured an available glaucoma ground truth. The top plot area features (1) the ROC curve (light green) with false positive rate and true positive rate on the x and y axis, (2) as well as the calibration curve (dark green) with mean predicted value and the fraction of positives on the x and y axis. A diagonal dotted black line between (0,0) and (1,1) indicates the ROC curve of random prediction and optimal calibration. The vertically flipped histogram of G-RISK predictions is aligned with the calibration curve in the bottom plot, with prediction value on the x axis, and prediction count on the y axis. Best viewed in color.

Figure 3 displays 12 multi-plots with ROC curve, calibration curve, and G-RISK prediction histogram per data set. Predictions fell between 0.2 and 1.0, with mode typically around 0.45 in sets with more than 5000 cases. Calibration curves seem to follow a sigmoidal shape, with a constant fraction of positives until a mean predicted value of 0.6 in the large data sets. Evaluation on ACRIMA provided the best calibrated predictions, with its calibration curve approaching the optimal dotted diagonal.

### Outperforming VCDR as a risk score

To better understand the differences between the G-RISK output and VCDR ground truth measured from the fundus image, both AUC values were computed for sets that contained a reliable VCDR. G-RISK outperformed VCDR in all five data (sub)sets, with an AUC disparity of 0.09 and 0.12 on REFUGE2 and RIM-ONE r3 data, respectively. AUC obtained with G-RISK did not differ significantly at an alpha of 0.05 (overlapping confidence intervals) in BMES and complete REFUGE1 data. This comparison is given in Table 3.

**Table 3:** Comparison of G-RISK performance versus image-measured VCDR as glaucoma detection proxy.

Data	#images	AUC G-RISK [95% CI]	AUC VCDR 1 [95% CI]	AUC VCDR 2 [95% CI]
BMES	6927	**0.967** [0.956–0.979]	0.958 [0.940–0.976]	NA
PAPILA (suspect referable)	488	**0.769** [0.722–0.815]	0.748 [0.699–0.798]	0.743 [0.691–0.795]
PAPILA (suspect non-referable)	488	**0.882** [0.840–0.923]	0.789 [0.728–0.851]	0.782 [0.716–0.847]
REFUGE1 test	400	**0.986** [0.974–0.999]	0.946 [0.907–0.984]	NA
REFUGE1 all	1200	**0.952** [0.925–0.979]	0.929 [0.902–0.956]	NA
REFUGE2 test	400	**0.867** [NA]	0.757 [0.693–0.815]	NA
RIM-ONE r3	159	**0.934** [0.889–0.978]	0.810 [0.723–0.897]	NA

VCDR was either retrieved from the cup and disc segmentation ground truth available (PAPILA, REFUGE data, RIM-ONE r3), or directly provided by the data set owners (BMES). For PAPILA, G-RISK results are compared against two independent human experts who segmented disc and cup. Best performance (AUC) per row is highlighted in bold text.

### Benchmarking against related work

Table 4 provides an overview of published reports on glaucoma detection with external testing. In principle, methods that used part of the data set for training were excluded from this comparison, as this presents an unfair advantage. The results on the REFUGE challenges are one exception to this. These data sets represent a prominent benchmark in the topic of glaucoma detection from fundus images. Therefore, a distinction is made between (1) pure external validation, and (2) trained on other parts of the same data set. G-RISK obtained the best results on ACRIMA (AUC = 0.88) and LAG (AUC = 0.93) data as external test sets reported in the literature. For REFUGE1 and REFUGE2, we limit the overview to the top five results. G-RISK would have obtained 2nd place on the 2018 REFUGE1 challenge, with a negligible difference in AUC with the best submitted result: only 0.003. The winning method relied on three models, while G-RISK only consists of one model. The second edition of REFUGE (2020) would have resulted in 3^rd^ place for G-RISK, with AUC 0.016 lower than the winning submission (not significant).

**Table 4:** Comparison of G-RISK performance with results in the literature that used the data sets as (external) test data to assess the generalizability.

Data	Method (challenge team)	Description	AUC [95% CI when available]
Pure external validation			
LAG	G-RISK	Proposed model	**0.93** [0.92–0.93]
	Fan et al. (2021)	Glaucoma detection CNN (binary) trained with OHTS fundus images	0.79 [0.78–0.81]
ACRIMA	G-RISK	Proposed model	**0.88** [0.86–0.91]
	Christopher et al. (2020)	Glaucoma detection CNN (binary) trained with fundus images from various private sources	0.86 [0.83–0.89]
	Diaz-Pinto et al. (2019)	Glaucoma detection CNN (binary) trained with various small publicly available data sets	0.77 [0.68–0.82]
	Fan et al. (2021)	Glaucoma detection CNN (binary) trained with OHTS data	0.74 [0.70–0.77]
Trained on other part of the same data set			
REFUGE1 test	Son et al. (2018)	Glaucoma detection CNN (binary), supplemented by CNN for glaucomatous disc changes, and CNN for RNFL defects	**0.989**
	G-RISK (pure external validation)	Proposed model	0.986
	SDSAIRC^a^	Glaucoma detection CNN (binary) fused with VCDR prediction from optic cup/disc segmentation using logistic regression	0.982
	CUHKMED^a^	Cup/disc segmentation CNN, derived VCDR values normalized as glaucoma prediction	0.964
	NKSG^a^	Glaucoma detection CNN (binary)	0.959
	VCDR ground truth	From official cup/disc segmentation labels	0.946
REFUGE2 test	Son et al. (2020)	Pretrained commercial system that was designed for 12 retinal aberrations	**0.883** [0.844–0.919]
	MIG^b^	Five glaucoma detection CNNs (binary), trained with fundus images from various small publicly available data sets, multi-scale input	0.876 [0.832–0.916]
	G-RISK (pure external validation)	Proposed model	0.867 [NA]
	MAI^b^	Glaucoma detection CNN with auxiliary task (test-time training^61^)	0.861 [0.816–0.904]
	Cheeron^b^	Glaucoma detection CNN with attention modules	0.856 [0.811–0.900]
	VCDR ground truth^b^	From official cup/disc segmentation labels	0.757 [0.693–0.815]

Best performance (AUC) per data set is highlighted in bold text.

^a^Team names, methods and AUC values retrieved from the REFUGE1 publication.

^b^Team names, methods and AUC values retrieved from the REFUGE2 publication.

### G-RISK output association with clinical metadata

The PAPILA data set allowed for assessing the association between G-RISK predictions and clinical metadata relevant to glaucoma. As shown in Table 5, only age and mean deviation (MD) of the 30–2 visual field exam were found to have a significant association. The results showed that as age increased, or MD became more severe, G-RISK predictions increased (Pearson correlation coefficient = 0.48 or −0.56, respectively). However, no significant association was found between G-RISK predictions and other metadata such as intra-ocular pressure, central corneal thickness, sex, and optical lens characteristics.

**Table 5:** Association between G-RISK predictions and clinical data in the PAPILA data set.

Clinical parameter	Pearson r [95% CI]	N° of data points
Age	0.48 [0.41; 0.55]	488
Sex	0.02 [−0.07; 0.11]	488
Dioptre 1	0.05 [−0.04; 0.14]	461
Dioptre 2	−0.04 [−0.13; 0.05]	480
Astigmatism	−0.01 [−0.10; 0.08]	479
Pseudophakic	0.05 [−0.04; 0.14]	477
IOP (pneumatic)	0.05 [−0.05; 0.14]	396
IOP (perkins)	−0.05 [−0.22; 0.12]	128
Pachymetry	0.06 [−0.03; 0.15]	474
Axial length	0.08 [−0.01; 0.17]	479
MD (30–2 exam)	−0.56 [−0.66; −0.45]	164

Pearson correlation coefficient, and number of data points are given per parameter. Parameter naming was based on the datasheet included in PAPILA.

### Glaucoma risk regression generalizes better than binary detection

A previously trained binary classification model with similar network architecture as G-RISK was evaluated on two selected test sets, REFUGE1 and BMES. The results showed that the binary classification model obtained an AUC of 0.87 [95% CI: 0.83–0.91] on REFUGE1, which was significantly lower than the AUC obtained using the G-RISK regression model (0.95 [95% CI: 0.93–0.98]). Similarly, on the BMES data, the binary classification model yielded an AUC of 0.76 [95% CI: 0.72–0.80], while the G-RISK model achieved an AUC of 0.97 [95% CI: 0.96–0.98]. These results confirm that the G-RISK model performed better than the binary classification model.

### Image processing pipeline for improved generalization

The importance of the 30° disc-centering procedure developed in this manuscript was investigated on REFUGE1 and AIROGS data. G-RISK performed worse, but still considered good, on the original 45° macula-centered images in both data sets: AUC dropped from 0.952 to 0.937 on REFUGE1, and from 0.972 to 0.921 on a subset of the AIROGS set. This result indicates that G-RISK is robust and can deal with macula-centered images with a larger FOV, while it never encountered this modality during training. An extreme zoom on ONH (crop factor of 0.4) led to drastic performance drops, falling to 0.840 and 0.764 in REFUGE1 and AIROGS, respectively. The absolute difference in AUC value following the normalization of all images to have a disc ratio of 0.23 or normalization by disc ratio computed per image dimension was not significant (differences of 0.003 and 0.004). Hence, natural heterogeneity in optic disc size may not affect G-RISK performance. The complete analysis is in Table 6.

**Table 6:** Effect of optic disc size correction during image preprocessing on G-RISK performance on REFUGE1 data and a random sample of AIROGS data (10%).

Image preprocessing approach	REFUGE1 2 camera types 45° macula-centered AUC [95% CI]	AIROGS 10% sample Multiple camera types 45° macula-centered AUC [95% CI]
Main method developed in this manuscript: 30° crop with disc ratio grouped per camera type/setting	0.952 [0.93–0.98]	**0.972** [0.97–0.98]
Original 45° image resized, no cropping	0.937 [0.91–0.97]	0.921 [0.91–0.93]
30° crop with standard crop factor of 0.65 (45° to 30° value)	0.952 [0.92–0.98]	0.961 [0.95–0.97]
Crop with random crop factor between 0.40 and 0.80	0.930 [0.90–0.96]	0.934 [0.92–0.95]
18° crop with crop factor of 0.40	0.840 [0.80–0.88]	0.764 [0.74–0.79]
30° crop with all optic discs rescaled to same size (=23% of image height)	**0.955** [0.93–0.98]	0.968 [0.96–0.97]

Best performance (AUC) per column is highlighted in bold text.

### Analysis of misclassified cases

**Fig. 4:**
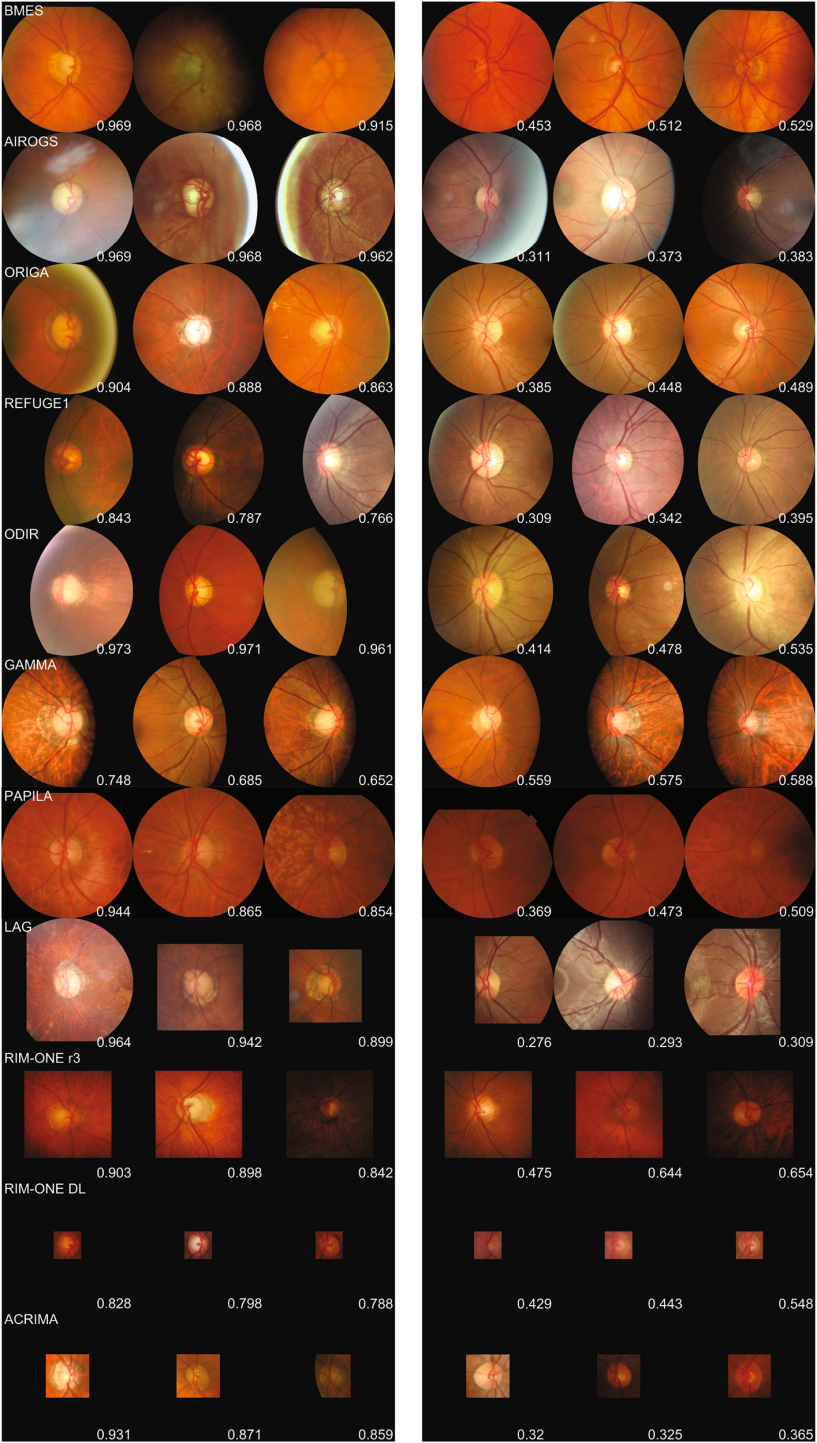
Overview of top three most extreme false-positive cases (three first images from the left per row) and false-negative cases (three rightmost images per row) per evaluated data set (name printed in the left corner of the first image per row). GHS data was left out as there exists no ground truth on image level. The predicted risk score is at the bottom right for each image. Best viewed in color and high resolution for optimal review by the reader. See Supplementary Fig. 3 for a view with overlaid saliency map.

All FP and FN, or a random selection if more than 20 cases exist, of the two population-based studies’ sets were reviewed by three glaucoma experts from three different countries. The number of FN was lower than 20 when thresholding at 0.70 for both sets. As a result, the total number of reviewed cases was equal to 33 and 27 for BMES and GHS data, respectively. Agreement between the reference standard available in both data sets and the majority vote of the independent glaucoma expert panel based on fundus images reading was only fair by a slight margin (κ = 0.217 and 0.229). Inter-rater agreement ranged from 0.104 to 0.335, indicating that there was little consensus on these misclassified cases. The consensus was higher on image quality scoring, with half of the comparisons achieving substantial agreement (κ between 0.61–0.80). The glaucoma expert review panel did seem to favor the inclusion of preprocessed images in their analysis. One expert even indicated that the preprocessed images were better for glaucoma diagnosis in all cases. This quantitative analysis is communicated in Table 7. Figure 4 is a composite image that features the top three most extreme FP and FN cases per evaluated data set. Supplementary Fig. 3 plots the same composite information with saliency maps overlaid for interpretability analysis. Recurrent features in extreme FP cases were extensive (non)physiological ONH cupping, visible lamina cribrosa, vessel bayoneting, vessel baring, peripapillary atrophy, and lack of visible RNFL bundles. For the FN cases, the most recurring pattern is noticeable localized RNFL defects in infero- and/or superotemporal sector, without matching glaucomatous ONH damage in the form of cupping/notching. One case featured a disc hemorrhage at the inferior sector. The number of FN cases was very limited in all evaluated data sets.

**Table 7:** Analysis of sampled misclassified images of BMES and GHS sets by three glaucoma expert graders, with additional analysis on image quality and image preprocessing impact.

	BMES (*n* = 33)	GHS (*n* = 27)
GLAUCOMA DETECTION Options: no glaucoma, suspect, definite	κ	κ
κ | G-RISK, majority vote experts (definite as referable)	−0.189	−0.143
κ | G-RISK, majority vote experts (suspect as referable)	0.004	−0.198
κ | Reference standard, majority vote experts (definite as referable)	0.217	0.229
κ | Reference standard, majority vote experts (suspect as referable)	−0.003	0.133
κ | Expert 1, Expert2 (definite as referable)	0.104	0.335
κ | Expert 1, Expert3 (definite as referable)	0.333	0.147
κ | Expert 2, Expert3 (definite as referable)	0.031	0.115
IMAGE QUALITY Options: ungradable, poor, good	κ	κ
κ | Expert 1, Expert2 (Good quality as positive class)	0.550	0.250
κ | Expert 1, Expert3 (Good quality as positive class)	0.672	0.658
κ | Expert 2, Expert3 (Good quality as positive class)	0.714	0.204
IMAGE PREPROCESSING AID Options: yes, no		
Expert 1 (proportion of cases where image processing had added value)	97%	89%
Expert 2 (proportion of cases where image processing had added value)	38%	50%
Expert 3 (proportion of cases where image processing had added value)	100%	100%

For glaucoma detection and image quality assessment, there were three options available as answer. Majority vote was defined as the agreement of at least two independent experts.

## Discussion

This study confirms the excellent performance of a trained CNN for glaucoma detection^14^ when applied to thirteen external data sets. To the best of our knowledge, this represents the greatest effort to date towards generalizability analysis by validating with data from two large population cohorts and eleven publicly available data sets. Moreover, results on the latter allow other researchers to benchmark their approach, an important aspect currently missing in research tackling glaucoma detection from fundus images. Given the wide variety of image types and glaucoma reference standards, we demonstrated the robustness of G-RISK for glaucoma risk prediction from color fundus images.

Evaluation on both BMES and GHS data resulted in an AUC of 0.976 and 0.984, respectively. At 95% specificity, sensitivities of 87.3% and 90.3% are obtained. This result satisfies the minimum criteria of 85% sensitivity and 95% specificity set by Prevent Blindness America^24^. For BMES, this represents a significant improvement over the screening results obtained using the Heidelberg Retina Tomograph (HRT), with specificity and sensitivity at 85.7% and 64.1% on participant level^25^. Of note, this comparison is not exact because the latter analysis was conducted on the ten-year follow-up data of BMES, with fewer participants than the population included in the present study. On AIROGS data, containing a diabetes population with realistic glaucoma prevalence, G-RISK obtained 80% sensitivity at 95% specificity, reaching the minimum requirements for human graders set by the data owners. G-RISK could have been used as a reliable grader during the labeling effort of more than 100,000 fundus images. Only a few studies described external validation on fundus images sourced from a population-based data set^26,27^. The glaucoma detection CNN by Liu and colleagues^26^ obtained an AUC of 0.964 on images from 6702 participants in the Handan Eye Study, of whom 2% had signs of glaucoma according to ISGEO criteria. Reported sensitivity and specificity were 91.0% and 92.5%. Their CNN was exposed to images captured by three different camera types during training, facilitating the extraction of domain-invariant features important to accurate glaucoma detection. The model evaluated in the current study did not leverage multi-source data at training time but still has excellent generalizability.

G-RISK achieved state-of-the-art results on publicly available data sets. Fan et al^28^. reported an AUC of 0.79 [95% CI: 0.78–0.81] on LAG data using a binary classification CNN trained using images of the Ocular Hypertension Treatment Study^29^, which is considerably lower than the 0.93 value in the present study. Christopher et al.^30^ reported an AUC of 0.86 [95% CI: 0.83–0.89] on ACRIMA data, which is two percentage points lower than the result of G-RISK. The glaucoma risk regression CNN would have obtained a second and third place on the two editions of the international REFUGE challenge^17^. This presents quite an achievement, as G-RISK did not train on part of that data, unlike the challenge participants. Training on part of a data set has the advantage that the model can familiarize itself with the data-specific imaging and ground truth characteristics. This advantage does not exist in prospective screening data.

The obtained excellent generalizability is predominantly due to the regression nature of the model. It could learn more about the continuous disease severity spectrum during training than with a standard binary classification approach. Empirical research pointed out that deep modeling with soft labels outperforms conventional classification CNNs in generalization and convergence speed^31^. Regression approaches have recently found their way in the field of semantic segmentation, a task that is conventionally achieved using pixel-based classification with hard labels. In semantic segmentation, the most uncertain areas are typically found at the edges of tissue delineation, where multiple domain experts may have different interpretations. The SoftSeg^32^ approach, introduced in 2020, addressed this issue by advocating for the use of soft labels (values between 0 and 1) at these edges to incorporate the label uncertainty. This approach has been shown to significantly improve the state-of-the-art on three medical imaging data sets by using a regression loss and linear activation. In the present study, G-RISK was able to select the most relevant domain-invariant features relevant to glaucoma detection due to a rich ground truth label, as well as a modeling framework that optimizes the learning of the information present in the label. G-RISK performance was also directly compared to a previously trained binary glaucoma detection model, featuring the exact same network architecture, except for the loss and final activation function. Extreme performance drops on REFUGE1 and BMES data of 0.08 and 0.21 in AUC values provide further evidence for the improved generalizability when training a CNN using soft labels.

The learning of domain-invariant features at the optic nerve head is highlighted by the performance on images that featured severe ONH crop in their original format. After scaling to a disc size that approaches the one found in 30° FOV images, G-RISK obtained excellent performance for glaucoma referral. Another proof of model robustness due to regression is the high performance on original data that features 45° FOV. AUC on original 45° REFUGE1 data was not significantly lower than the AUC obtained on preprocessed disc-centered 30° images. On AIROGS data, the difference was significant, probably due to the more extensive heterogeneity in image types present in the data set. That is where the proposed image processing pipeline can improve the performance even more. By minimizing the shift between training images and external testing images, the risk of faulty predictions due to outliers or OoD data is reduced.

Regression approaches in the context of glaucoma imaging have been described previously^13,22,27^. Medeiros et al.^13^ introduced Machine-to-Machine (M2M), a type of regression CNN that estimates the average OCT-measured circumpapillary RNFL thickness from disc-centered fundus images as a proxy for neural loss. Pearson’s correlation coefficient between OCT-measured ground truth and prediction was 0.83. In follow-up studies, their research group revealed that M2M could discriminate glaucoma in a population-based screening program in Brazil. It may outperform human experts in detecting eyes with repeatable visual field loss^33,34^. It would be interesting to see M2M’s performance on some publicly available data, to get an idea of how it benchmarks with related work. Although average RNFL thickness may be an objective parameter to quantify neural damage, it also has its imperfections. First, wrong RNFL segmentation or anatomical variants can lead to exams being labeled as potentially abnormal and an erroneous glaucoma diagnosis. This ‘red disease phenomenon’ is well-known among OCT users, and OCT results should therefore be carefully reviewed by a glaucoma expert, which introduces human subjectivity. Next, RNFL thinning is no pathognomonic sign of glaucoma only^35^. RNFL defects have been associated with other ocular^36,37^ and systemic^38^ diseases. Although such cases should be referred to an ophthalmologist anyhow, it might be unclear whether RNFL defects without matching ONH damage indicates the presence of glaucoma or a different condition.

Recent research has also investigated deep learning approaches for the joint segmentation of the optic cup and disc from fundus images. This includes implementing various modifications to the U-Net architecture^39^, which have yielded competitive results on both tasks. By segmenting the optic cup and disc, VCDR values can be derived and calculated. However, relatively few published segmentation approaches have specifically evaluated the generalizability of glaucoma detection. The REFUGE1 participating team CUHKMED obtained 3rd place when thresholding the segmentation-based VCDR against the glaucoma ground truth as reported in Table 4. Additionally, Fu et al.^40^ tested a VCDR prediction from a polar-transformed fundus image externally on 1676 fundus photos of the Singapore Chinese Eye Study (SCES), reporting a competitive AUC of 0.90. However, since SCES data is not publicly available, no benchmarking was possible within the current study. In contrast, G-RISK takes a different approach by directly estimating the VCDR from the fundus image without the need for segmentation, bypassing the need for joint segmentation of the optic cup and disc. Similarly, Alipahani et al.^27^ recently developed a regression CNN that directly estimates VCDR from fundus images in AI-based phenotyping of ONH morphology. Pearson’s correlation coefficient between VCDR ground truth and prediction was 0.89 on a small subset of UK Biobank fundus images. Their approach obtained an AUC of 0.76 [95% CI: 0.74–0.78] when thresholding the VCDR prediction against a glaucoma label based on patient self-reporting and codes of the International Classification of Diseases (ICD). While we do not report on UK Biobank data, it is worthwhile to explore any performance differences between G-RISK and the model developed by Alipahani et al. Moderate AUC values for glaucoma detection could be caused by a weak ground truth, as self-reporting is likely to be associated with the lower limit of 50% undetected cases present in the general population. The difference in methodology resides in the ground truth during model development, as G-RISK relied on VCDR estimation during ophthalmoscopy, while Alipahani et al. measured the ground truth from the images directly. Their research also pinpointed the strong association between VCDR and glaucoma risk, reporting a correlation of 0.91. G-RISK comprises more than VCDR estimation, backed by an analysis on five sets that objectively proves that G-RISK predictions outperform image-measured VCDR as a proxy for glaucoma risk. Furthermore, analysis on PAPILA clinical data suggests that G-RISK correlates well with glaucoma. Both G-RISK and glaucoma exhibit a significant association with age and visual field defects, while only weak correlations exist with factors such as sex, and central corneal thickness outside of intraocular pressure measurements. Optical lens characteristics are known to have no association with glaucoma.

Powerful disease detection algorithms should have calibrated predictions^41^, a characteristic in which the prediction is representative of the disease likelihood. Conventional classification CNNs with sigmoid activation are known to be poorly calibrated^42^. The G-RISK prediction value can be interpreted as a risk score between 0.2 and 1. Up until values around 0.7, G-RISK overpredicts (calibration curve below the optimal calibration line). Data sets with a prevalence lower than 10% follow the same calibration curve. A uniform calibration operation might lead to transformed predictions between 0 and 1 that can be interpreted as calibrated glaucoma risk across population data. This exercise was out of the scope of the current study but will be covered in the future.

The explainability of the G-RISK model was evaluated through two setups. Firstly, a thorough examination of the most extreme false positive (FP) and false negative (FN) cases by glaucoma experts revealed instances with large (non-)physiological optic nerve head cupping, peripapillary atrophy, and missing RNFL bundles for the FP group. On the other hand, FN cases featured repeated RNFL defects without corresponding ONH damage. In addition to manual expert analysis, Supplementary Fig. 3 presents the same FP and FN images, overlaid by salient maps generated using gradient analysis. Individual inspection did not reveal a recurrent salient region. For more information on CNN-based glaucoma detection from fundus images and objective explainability analysis, the reader is referred to our previous work^14^. In the latter, Fig. 3, second row, first image on the left, illustrates recurrent saliency patterns obtained by averaging over a test set of more than 4000 fundus images. The saliency of G-RISK is concentrated in the infero- and supero-temporal areas inside and outside of the ONH.

This study advances the research area of generalizable glaucoma detection CNNs through the external testing on population cohorts and heterogeneous publicly available data. There are still important knowledge gaps left. Using a fixed threshold value did not result in consistent specificity values across the 13 data sets (ranging from 0.70 to 0.99). Therefore, further model calibration is necessary to achieve uniform sensitivity and specificity levels. It is worth noting that the heterogeneity of the glaucoma ground truth definition also plays a significant role in this behavior. Next, the two population cohorts feature people with predominantly European ancestry (Germany and Australia). Hence, generalization on screening populations from other ethnic backgrounds is unknown. Still, performance remained high on publicly available data collected in countries such as China and Singapore, but feature prevalence levels higher than in the general population. In addition, performance across glaucoma severity was not assessed, as these labels were not available in the data sets. One exception is GAMMA, on which G-RISK obtained an excellent AUC of 0.99 in the early glaucoma class. Finally, G-RISK fails in rare cases with subtle RNFL defects or disc hemorrhages without matching ONH damage. Future updates aim to implement changes that decrease the false-negative rate further.

The strengths of this study are significant. First, we addressed the issue of generalizability in fundus-based glaucoma detection models through extensive validation on thirteen external sets, totaling 149455 images. We tackled a significant challenge because the data sets have considerable heterogeneity in glaucoma ground truth, camera type, and population type. Next, we analyzed the influence of factors such as natural ONH size variability and image scale. Results were benchmarked against the literature, highlighting state-of-the-art performance by G-RISK. We demonstrated that G-RISK was trained on VCDR estimates from ophthalmoscopy but performs better than image-measured VCDR in the task of glaucoma referral.

Excellent generalizability of AI-based glaucoma detection from fundus images has been demonstrated in this work, both on large screening sets and various publicly available data sets. In retrospective glaucoma screening, G-RISK complies with the minimum requirements set by Prevent Blindness America. Further validation of G-RISK using prospective studies is warranted.

## Methods

### Study design

This study adheres to the STARD 2015 guidelines for the standardized reporting of evaluation of a diagnostic test, as well as to the tenets of the Declaration of Helsinki. The training material for G-RISK was retrospectively collected from the University Hospitals Leuven, and approved by the Ethics Committee Research UZ / KU Leuven under study number S60649. Informed consent was waived due to the retrospective nature of the research project, and all fundus images were deidentified before use. For informed consent of the data used for external testing, we refer to the administrators of the respective data sets.

### Study population—Model development

Glaucoma detection was achieved using a custom ResNet-50^43^ CNN model described in our previous work^14^ that focused on the explainability of the CNN in two glaucoma applications. In that study, 23,930 stereoscopic fundus images (12,265 eyes, 6486 individuals) were selected for training, validation, and internal testing. Fundus images were captured at the glaucoma department of the University Hospitals Leuven (UZL), Belgium, between 2010 and 2018. Hence, the majority of images feature signs of glaucoma. Inclusion criteria for this set were the availability of a matching 30° fundus photo (imaged with a Zeiss VISUCAM® at 1620 × 1444). Glaucoma was based on evaluation by a glaucoma expert using perimetry, IOP, fundoscopy, and retinal imaging. This clinical evaluation included VCDR estimation during fundoscopy, which was selected as the reference risk label during G-RISK development. This continuous value between 0 and 1 was thresholded against a binary glaucoma ground truth to obtain glaucoma detection results. The benefits of using a continuous versus a binary target variable are well-studied in the literature under soft labels. In glaucoma detection, an approach with soft labels allows the model to leverage the richer information of expert annotations during training. The CNN can grasp differences in disease severity, going from no cupping to an optic nerve that has completely cupped. In binary detection, both early symptoms (e.g. RNFL defect, notching, vessel baring) and extreme cupping are bundled in the glaucoma category, which does not accommodate the learning of intermediary severities. To quantify the improved generalizability when using a regression approach, we also validated a binary classification CNN for glaucoma detection on two test sets. This CNN was trained in a similar setup, with the only changes in the glaucoma ground truth (defined by glaucoma expert based on multimodal exam), cross-entropy as loss function instead of mean squared error, and sigmoid activation instead of a linear activation at the end of the ResNet-50 architecture. It was described in detail in our previous work^14^.

### Study population—Model testing (external validation)

We evaluated our model using fundus images from two major population studies and eleven publicly available data sets. External fundus image data sets were eligible for evaluation given the following conditions: (1) availability of a (suspected) glaucoma label, and (2) majority (>50%) of images containing the optic nerve head (ONH). Both the imaging protocol and the definition of glaucoma varied considerably across the test sets.

#### BMES

The Blue Mountains Eye Study (BMES) is a large population-based study for ocular diseases held three decades ago in an urban area in Australia^2^. 3654 individuals aged 49 or older participated in the eye examination from 1992–1994. Fundus images were captured using an analog Zeiss FF3 film camera with subsequent digitization of the images. Open-angle glaucoma (OAG) was diagnosed in case of (1) visual field loss of Humphrey Field Analyzer 30–2 exam, (2) matching neuroretinal rim thinning, (3) VCDR exceeding or equal to 0.7, (4) asymmetric cupping between eyes (>0.3), (5) and when gonioscopic results indicated no angle closure.

#### GHS

The Gutenberg Health Study (GHS) is a large population-based study held in mid-western Germany, with a baseline encompassing 15010 participants between 35 and 74 years^23^. 30° optic disc-centered images were collected using a Zeiss VISUCAM fundus camera. Glaucoma diagnosis was established using a modification of the International Society for Geographic and Epidemiological Ophthalmology (ISGEO) guidelines including disc size adjustment^44^. Final grading considered VCDR, asymmetric cupping between eyes, and rim narrowing (<10% of the corresponding disc diameter). ISGEO grading was available for at least one eye in 12089 individuals examined at baseline.

#### AIROGS

The Rotterdam EyePACS AIROGS data set consists of 113893 fundus images of 60357 individuals who visited numerous centers of the EyePACS network in the United States^45–47^. The training set of 101442 images was made available in late 2021 in the context of an international challenge on glaucoma detection from fundus images. The optic discs in the fundus images were assessed by a team of 22 glaucoma experts (at least two graders per image), who had at least a sensitivity of 80% and a specificity of 95%. Referable glaucoma was defined using ten structural features or biomarkers, and when the annotator expected corresponding visual field damage.

#### ORIGA

The Online Retinal Fundus Image Database for Glaucoma Analysis and Research (ORIGA) contains 650 randomly selected images from the Singapore Malay Eye Study (SiMES), a population-based study conducted between 2004 and 2007^48^. The glaucoma labeling procedure was not defined. Images were captured at a wider angle than 30° using an unspecified camera device.

#### REFUGE1

The Retinal Fundus Glaucoma Challenge (REFUGE) was held at MICCAI 2018, to provide a unified evaluation framework for objective comparison of glaucoma detection models using fundus images^49^. 400 images were captured with a Zeiss VISUCAM, the remaining 800 with a Canon CR-2 of a glaucoma clinic located in China. All images are macula-centered at a 45° viewing angle. The glaucoma reference standard was obtained after a multimodal assessment of clinical records, including IOP, OCT, visual fields, and follow-up examinations. 120 cases of the data set are glaucomatous (POAG or NTG), representing 10% of the data.

#### LAG data

The large-scale attention-based glaucoma detection database (LAG) consists of 4854 fundus images sourced from a Chinese hospital^16^. The reference standard was established using IOP, visual field exams, and manual ONH assessment by qualified specialists. Glaucoma was diagnosed in 1711 images, representing 35% of the data set. All images contain a visible ONH and were captured using an unspecified mix of fundus cameras at varying angles. Given the inconsistent image-altering procedure the data set creators used, it is impossible to use the disc ratio as a proxy for correct 30° cropping.

#### ODIR

The Ocular Disease Intelligent Recognition (ODIR) challenge was organized in 2019 to stimulate research on multi-disease classification from fundus images^50^. The complete set encompasses 10000 images from 5000 patients (one image per eye), of which 7000 are currently available to download. Macula-centered images were captured using different devices from manufacturers such as Canon, Zeiss, and Kowa. Next to glaucoma cases (4.7%), expert-annotated labels exist for diabetic retinopathy, cataract, age-related macular degeneration, hypertension, and myopia.

#### REFUGE2

Following the successes of the first REFUGE challenge in 2018^17^, the organizers organized a second edition as part of MICCAI 2020^49^. In a similar setup, 800 additional images were added to the data set. The new fundus images had been acquired using fundus cameras manufactured by Kowa (validation) and Topcon (test).

#### GAMMA

The Glaucoma Grading from Multi-Modality Images (GAMMA) challenge invited participants to develop and validate models for glaucoma detection using fundus images and OCT scans^51^. The available training data contains 50 non-glaucoma cases, 25 cases with early glaucoma, and 25 cases featuring mild or advanced glaucoma. Similar to REFUGE data, specialists assigned the glaucoma reference standard based on fundus photography, IOP, VF, and OCT.

#### RIM-ONEr3

The Retinal IMage databases for Optic Nerve Evaluation (RIM-ONE), first shared in 2011, were initially intended to evaluate algorithms for optic disc segmentation^52^. The third revision in 2015 contains 85 images of healthy eyes and 74 images of glaucoma patients. Images were captured using a Kowa WX 3D stereo fundus camera at a single center in Spain. The FOV spans 20° horizontally and 27° vertically.

#### RIM-ONE DL

Launched in 2020, the creators of RIM-ONE data sets updated their fundus images to evaluate deep learning algorithms for glaucoma detection^53^. All of the images were re-evaluated by two experts and originated from different hospitals, captured with different cameras. The total set encompasses 313 non-glaucoma fundus images and 172 fundus images with confirmed glaucoma (photo evaluation by glaucoma expert). The images are characterized by standardized cropping operation around the optic disc.

#### ACRIMA

In total, 705 images of the ACRIMA project, founded by the government of Spain for automated retinal disease assessment, were made available in 2019^54^. Images were captured with a Topcon TRC fundus camera at a 35° FOV. Images were labeled for glaucoma by two experts and cropped around the optic disc using a bounding box of 1.5× the optic disc radius. Notably, the glaucoma images are characterized by a larger image size than the non-glaucoma images.

#### PAPILA

Recently made available to the research community, PAPILA is the first data set providing color fundus images and clinical data of both eyes of the same study participant. Being able to use the joint information of both eyes for glaucoma detection approaches real-life screening scenarios. PAPILA consists of 488 fundus images belonging to 244 individuals, captured with a non-mydriatic Topcon TRC-NW400 device with an FOV of 30°. The glaucoma ground truth label is presented in three categories: glaucomatous, non-glaucomatous, and suspect, based on the evaluation of clinical data by trained ophthalmologists. All images contain the optic disc, with expert segmentation of disc and cup provided.

### Image quality control

Image quality was assessed through the segmentation of the ONH using a generalizable CNN developed and validated^14^. In case of availability of a ground truth ONH segmentation mask in the data set, this step was skipped (ORIGA, REFUGE1, GAMMA, RIM-ONEr3, and PAPILA). The CNN-generated optic disc segmentation image was tested against two criteria for a realistic optic disc. First, the vertical optic disc size per object candidate in the segmentation image was divided by the image height to obtain a disc ratio. This disc ratio should be between 0.10 and 0.40 for images with a FOV of at least 30°. Next, the optic disc candidate was selected based on the first central Hu moment^55^, a value invariant to the transformation that equals 0.159 when the shape is a perfect circle. The candidate with the Hu moment closest to 0.159 was selected to discard oblong non-circular segmented objects. The image was discarded from the analysis if no candidate matched the criteria. There was no human intervention in this automated process. Supplementary Fig. 1 describes the removal rate per data set.

### Image transformation to 30° disc-centered fundus image: original FOV exceeding 30°

Each image with a CNN-detected or human-verified optic disc underwent multiple processing steps to minimize the covariate shift between the external and original training data. First, the image underwent a 30° cropping/extension operation centered on the localized optic disc following ONH segmentation. Original FOV per data set could be determined based on the optic disc size concerning the vertical image dimension (disc ratio) or through the information present in the data set description. In the development set, which contains exclusively 30° disc-centered images, the disc ratio was equal to 0.23 averaged over 23930 images.

$${crop}\,{factor}=\,\frac{{discrati}{o}_{{original}}}{{discrati}{o}_{30^\circ }}=\frac{{discrati}{o}_{{original}}}{0.23}$$

Disc ratios were averaged per image size per data set. For a data set with fundus images featuring a 45° FOV, the average disc ratio will be around 0.15, which would imply a crop factor of 0.65. Using a uniform crop factor per data set is essential, as crop factor per image would remove the natural heterogeneity in optic disc size. Two data sets (ACRIMA, LAG) made it impossible to preserve this normal variation due to the cropping procedure already present in the original data. Therefore, they are marked with an asterisk in the results table. In data sets that feature multiple image sizes (AIROGS, ODIR, REFUGE1, REFUGE2), disc ratios were averaged per image size and set to the global data set average if there were less than ten cases of specific image size. The crop factor was multiplied by the vertical image size to obtain a 30° disc-centered image. Zero padding was applied to the cropped image if the disc-centered crop exceeded the original image boundaries in a specific direction, as can be expected in macula-centered images where the ONH is situated at the image border. We analyzed the importance of the proposed 30° disc-centered image cropping through a sensitivity analysis on REFUGE1 data and a random 10% subset of AIROGS data. These sets feature multiple image dimensions, next to a well-defined glaucoma label.

### Image transformation to 30° disc-centered fundus image: original FOV smaller than 30°

Some data sets feature images with smaller FOV values (RIM-ONE r3, LAG), or were cropped around the optic disc (ACRIMA, RIM-ONE DL). Image extension or padding was applied to ensure correct optic disc scale and lighting correction in this case. This was done by copying the original image’s border value in both height and width directions until the average disc ratio equals 0.23. After lighting correction, the image area with copied value (synthetic image information) was replaced by black pixels prior to G-RISK evaluation. See Supplementary Fig. 2 for an example of the proposed image extension procedure.

### Further processing

Processed images were subjected to a filtering operation to counter unequal lighting due to the curvature of the retina^56^. Finally, images were resized to 512 × 512 and 3 RGB color channels, and divided by 255 to match the input requirements of the trained G-RISK model. All image operations per data set are explained and visualized in detail in Supplementary Fig. 2.

### Evaluation procedure

All predictions by the G-RISK were evaluated against the reference glaucoma label using thresholding. The area under the receiver operating characteristic (ROC) curve (AUC) was selected as the primary performance metric, accompanied by balanced sensitivity and specificity by minimizing the difference between the two. Harmonized sensitivity and specificity was selected as the costs associated with FP and FN can vary depending on the deployment setting. For the three data sets that featured a prevalence that approaches general population scenarios (BMES, GHS, and AIROGS), additional sensitivities were reported at 90%, 95%, and 97.5% levels of specificity. This choice was motivated by the importance of specificity in the context of glaucoma screening. There exists a general consensus that specificity should be as high as possible, to prevent a large inflow of individuals who do not actually have the disease. Additionally, predictions were thresholded at a fixed value of 0.7 to assess glaucoma detection performance uniformly across data sets. 0.7 was selected as this is a common VCDR threshold for glaucoma detection. Evaluation was also conducted on participant level for the two population cohorts (BMES and GHS) and publicly available PAPILA set, as glaucomatous damage can be unilateral in a glaucoma patient. In order to mimic expert referral as closely as possible, the maximum predicted risk score of the two eyes (when available) was evaluated against the reference standard. 95% confidence intervals for AUC were computed using fast DeLong’s algorithm^57^. All statistical analyses were performed using the SciPy Python library^58^. One exception to this is REFUGE2, for which the reference standard is currently not accessible to researchers. The AUC value for this set was retrieved from the online evaluation server hosted by the challenge organizers and through direct e-mail communication. For data sets that contained a VCDR ground truth label (REFUGE1, BMES, RIM-ONEr3, REFUGE2 test set, and PAPILA), we compared the performance of G-RISK with VCDR by thresholding the VCDR variable against the glaucoma ground truth. Furthermore, we report on the association between G-RISK predictions and clinical metadata including IOP, mean deviation of the visual field (MD), axial length, refractive error, and corneal thickness using the PAPILA data set. ROC curves were complemented with a calibration curve (10 bins)^59^ and the histogram of predictions in the same plot. Results from related work on deep learning-based glaucoma detection and generalizability were included to compare where possible (LAG, ACRIMA, REFUGE1 test set, REFUGE2 test set). To better understand the decision-making process of G-RISK, three independent glaucoma experts manually evaluated randomly selected false positives (*n* = 20) and false negatives (*n* = 20) of both the BMES and GHS data. In case there were less than 20 cases, the total number of FP or FN were analyzed. Expert graders assessed image quality (good, poor, bad), glaucoma (no, suspect, definite), listed the reasons for glaucoma diagnosis, and indicated whether the processed image aided in their diagnosis. Cohen’s kappa coefficient (κ) assessed inter-grader agreement and agreement with glaucoma ground truth. The three most extreme FP and FN for all data sets were plotted (with and without overlaid saliency map) with accessible ground truth label and images. Saliency maps were generated using the gradient method provided by the iNNvestigate library v2.0.1^60^.

## Supplementary information

Supplementary Material

## Data Availability

Data used were sourced from both private and publicly available data sets. Publicly available data (AIROGS, ORIGA, REFUGE1, LAG, ODIR, REFUGE2, GAMMA, RIM-ONEr3, RIM-ONE DL, ACRIMA, and PAPILA) can be retrieved from the source website. For the testing on BMES and GHS data, the authors established a scientific research collaboration with the data owners in Sydney and Mainz, respectively. Images and ground truth cannot be shared by the authors directly; we invite interested scientists to contact the original owners of the various data sets. The predictions generated by G-RISK on the publicly available data sets could be shared upon reasonable request (e-mail: ruben.hemelings@kuleuven.be). GHS data: “Written informed consent from GHS study participants does not allow public access to the data. Access to the data in the local database is possible at any time upon request according to the ethics vote. This concept was developed with the local data protection officer and the ethics committee (local ethics committee of the Rhineland-Palatinate Medical Association, Germany). Interested scientists can make their requests to the Gutenberg Health Study Steering Committee (e-mail: info@ghs-mainz.de).”

## Code availability

The standard ResNet-50 code used in this study is available at https://keras.io/api/applications/resnet/#resnet50-function. The evaluation script to obtain the predictions on the different data sets are available upon academically reasonable request (e-mail: ruben.hemelings@kuleuven.be). All of the code was developed and carried out in a kernel with Python version 3.8.12 on a Windows 10 machine.
