# Supplementary material for: A generalizable deep learning regression model for automated glaucoma screening from fundus images
Source: NPJ Digit Med. 2023 Jun 13;6:112. doi: 10.1038/s41746-023-00857-0 (PMC10264390; doi:10.1038/s41746-023-00857-0)

Supplementary Material

Supplementary Figure 1 – images qualified for G-RISK analysis

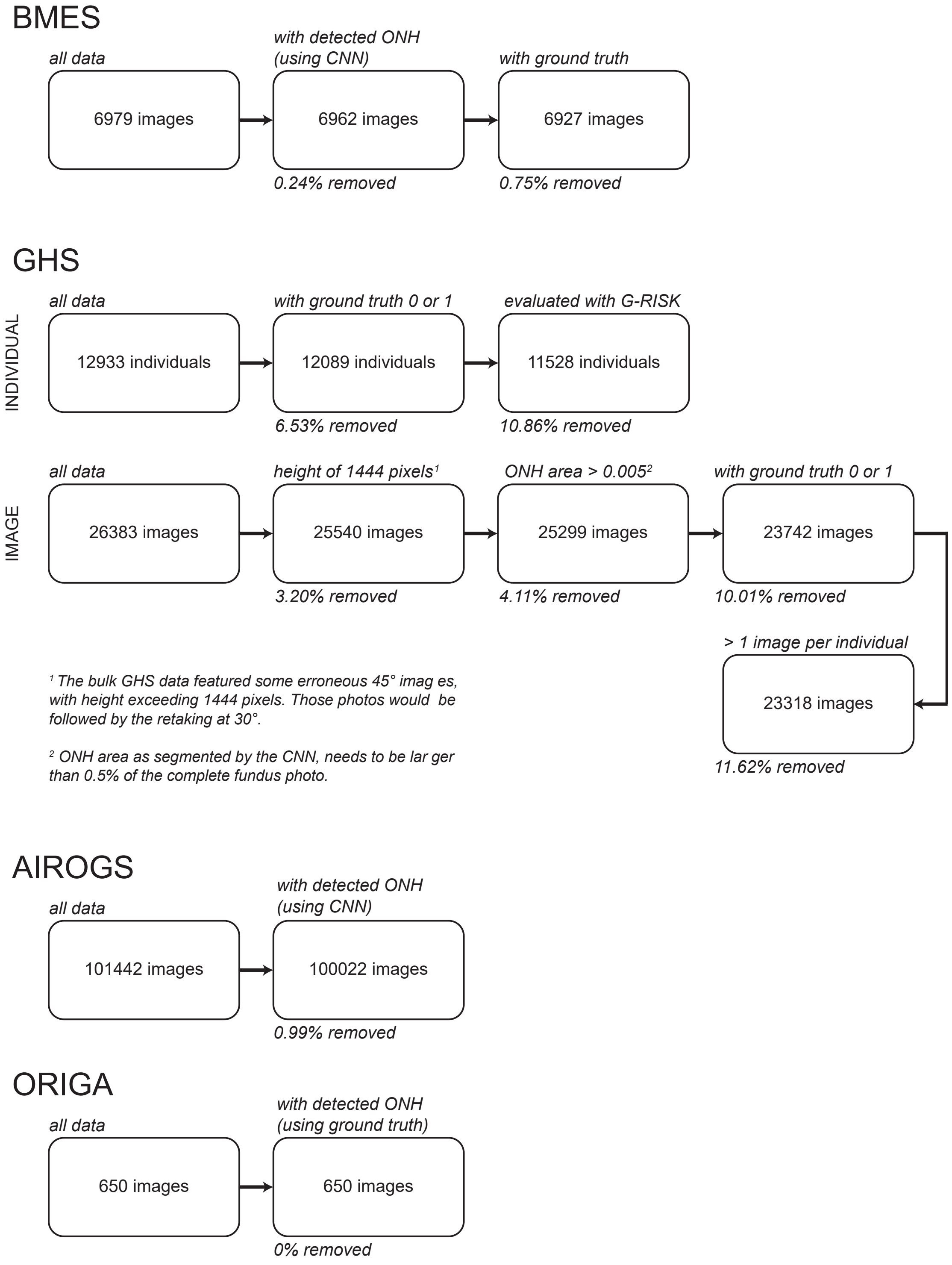

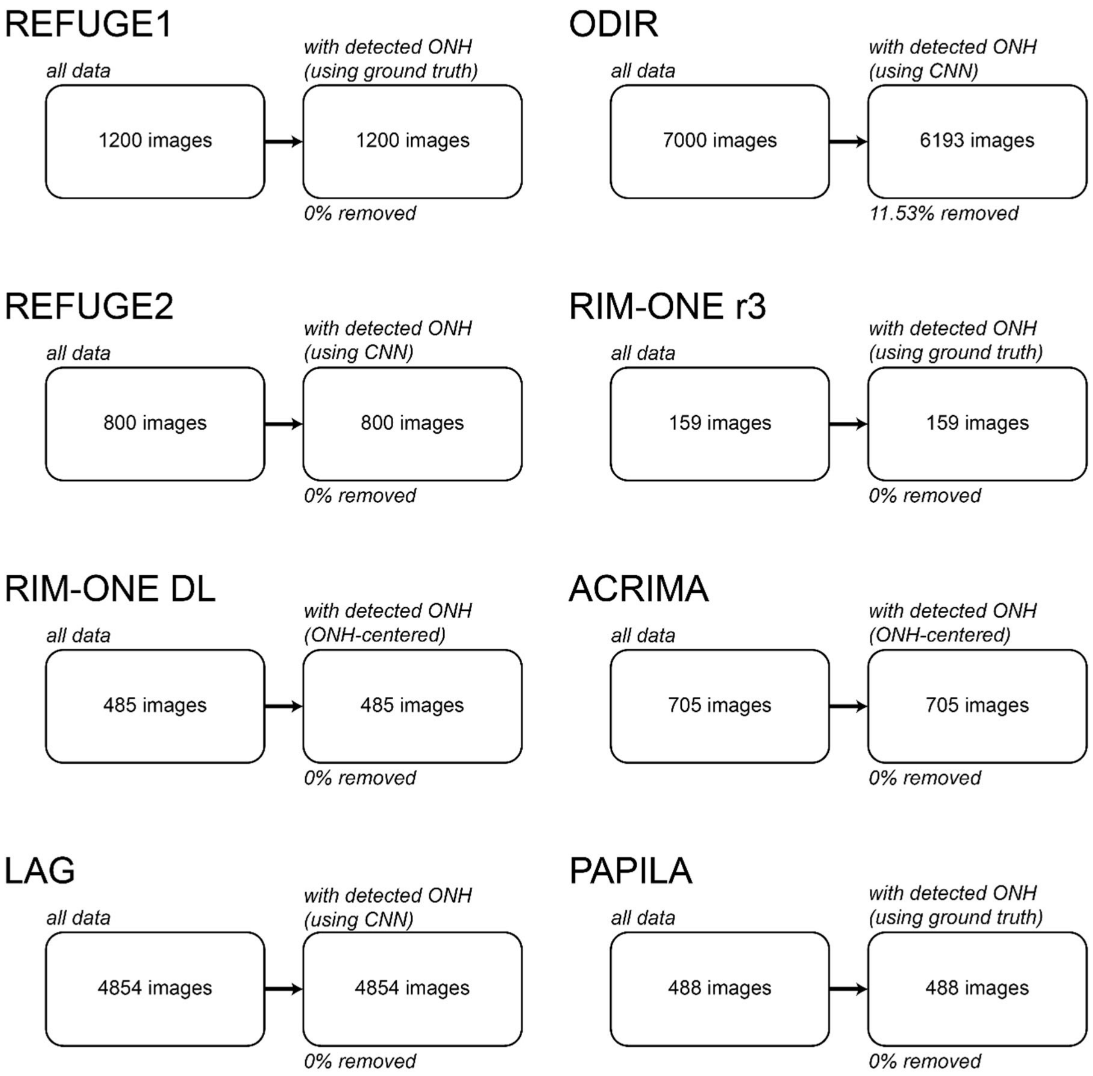
REFUGE1
all data
1200 images
with detected ONH (using ground truth)
1200 images
0% removed
ODIR
all data
7000 images
with detected ONH (using CNN)
6193 images
11.53% removed
REFUGE2
all data
800 images
with detected ONH (using CNN)
800 images
0% removed
RIM-ONE r3
all data
159 images
with detected ONH (using ground truth)
159 images
0% removed
RIM-ONE DL
all data
485 images
with detected ONH (ONH-centered)
485 images
0% removed
ACRIMA
all data
705 images
with detected ONH (ONH-centered)
705 images
0% removed
LAG
all data
4854 images
with detected ONH (using CNN)
4854 images
0% removed
PAPILA
all data
488 images
with detected ONH (using ground truth)
488 images
0% removed

Supplementary Figure 2 – image processing pipeline per data set

BMES

BMES fundus images are centered on the ONH, and were photographed at an FOV of 35°.

Transformation to 30° disc-centered image: **CROPPING**

Image	Processing step	Motivation
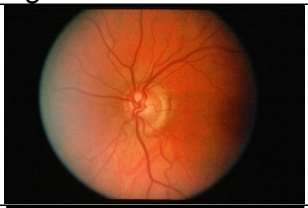	Original image	
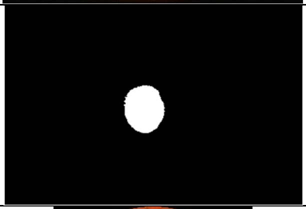	ONH localization	Required for cropping of 30° FOV image with centering on the ONH, in addition to the image quality control.
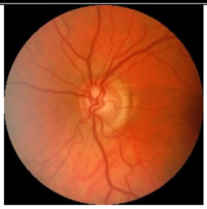	30° cropping with ONH centered	Disc ratio grouped by image dimensions was divided by 0.23 (average disc ratio of 30° fundus image in G-RISK training data)
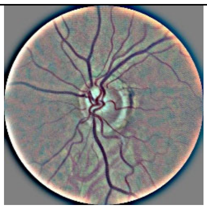	Lighting equalization	Lighting equalization, typically used in larger fundus images with larger FOV, was applied to approach the color distribution of G-RISK training images
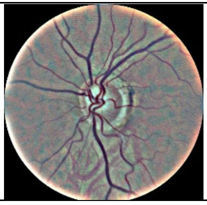	Circle crop with radius 1	To mimic the circular FOV encountered typically in 30° images, pixels outside the circle are replaced by a black color.

GHS

GHS fundus images are centered on the ONH, and were photographed at an FOV of 30°.

Transformation to 30° disc-centered image: **NO ACTION NEEDED**

Image	Processing step	Motivation
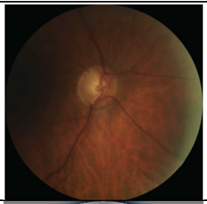	Original image	
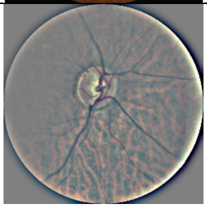	Lighting equalization	Lighting equalization, typically used in larger fundus images with larger FOV, was applied to approach the color distribution of G-RISK training images
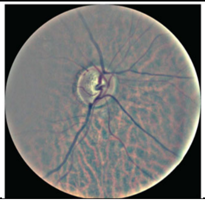	Circle crop with radius 1	To mimic the circular FOV encountered typically in 30° images, pixels outside the circle are replaced by a black color.

AIROGS

AIROGS fundus images do not all contain a detected ONH, and were photographed at varying FOV.

Transformation to 30° disc-centered image: **CROPPING/EXTENSION**

Image	Processing step	Motivation
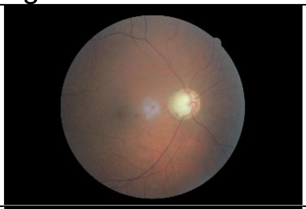	Original image	
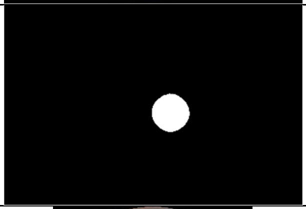	ONH localization	Required for cropping of 30° FOV image with centering on the ONH, in addition to the image quality control.
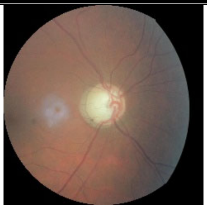	30° cropping with ONH centered	Disc ratio grouped by image dimensions was divided by 0.23 (average disc ratio of 30° fundus image in G-RISK training data). Crop factor was between 0.45 and 1.30
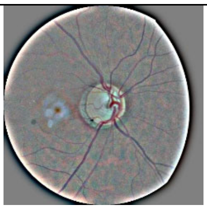	Lighting equalization	Lighting equalization, typically used in larger fundus images with larger FOV, was applied to approach the color distribution of G-RISK training images
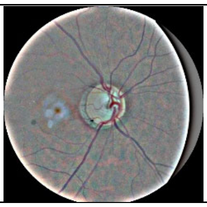	Circle crop with radius 1	To mimic the circular FOV encountered typically in 30° images, pixels outside the circle are replaced by a black color.

PAPILA

PAPILA fundus images are centered on the ONH, and were photographed at an FOV of 30°. However, the FOV shape seems different compared to the 30° FOV in the training images.

Transformation to 30° disc-centered image: **CROPPING**

Image	Processing step	Motivation
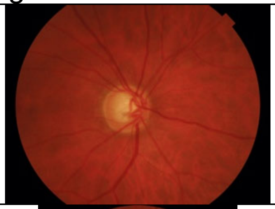	Original image	
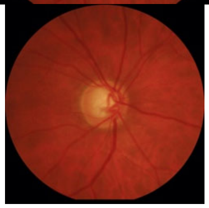	30° cropping with ONH centered	Disc ratio over complete PAPILA was close to 0.25, implying a smaller FOV when compared to the 30° UZL training images. Crop factor was 0.25/0.23 = 1.09
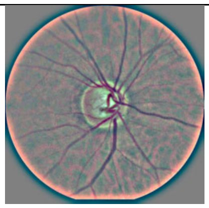	Lighting equalization	Lighting equalization, typically used in larger fundus images with larger FOV, was applied to approach the color distribution of G-RISK training images
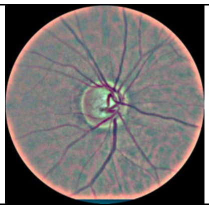	Circle crop with radius 1	To mimic the circular FOV encountered typically in 30° images, pixels outside the circle are replaced by a black color.

ORIGA

ORIGA fundus images all contain a visible ONH, and were photographed at an angle exceeding 30°.

Transformation to 30° disc-centered image: **CROPPING**

Image	Processing step	Motivation
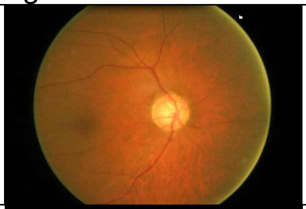	Original image	
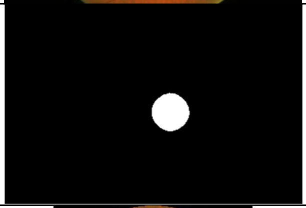	ONH localization	Required for cropping of 30° FOV image with centering on the ONH
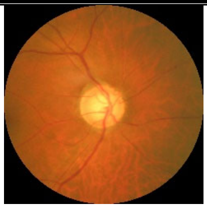	30° cropping with ONH centered	Disc ratio grouped by image dimensions was divided by 0.23 (average disc ratio of 30° fundus image in G-RISK training data), crop factor = 0.84
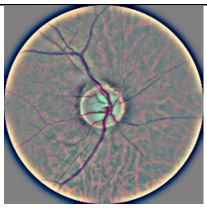	Lighting equalization	Lighting equalization, typically used in larger fundus images with larger FOV, was applied to approach the color distribution of G-RISK training images
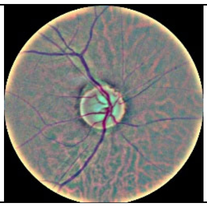	Circle crop with radius 1	To mimic the circular FOV encountered typically in 30° images, pixels outside the circle are replaced by a black color.

REFUGE1

REFUGE1 fundus images all feature a detected ONH, and were photographed at a 45° angle.

Transformation to 30° disc-centered image: **CROPPING**

Image	Processing step	Motivation
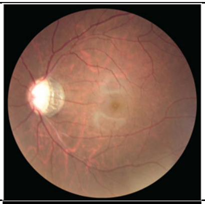	Original image	
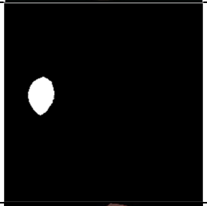	ONH localization	Required for cropping of 30° FOV image with centering on the ONH
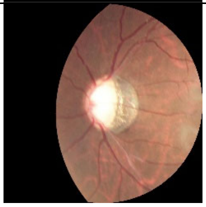	30° cropping with ONH centered	Disc ratio grouped by image dimensions was divided by 0.23 (average disc ratio of 30° fundus image in G-RISK training data). Crop factor = 0.69
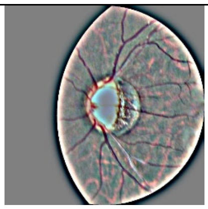	Lighting equalization	Lighting equalization, typically used in larger fundus images with larger FOV, was applied to approach the color distribution of G-RISK training images
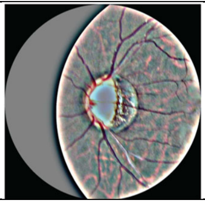	Circle crop with radius 1	To mimic the circular FOV encountered typically in 30° images, pixels outside the circle are replaced by a black color.

LAG

LAG fundus images all feature a detected ONH, but images vary in FOV (both greater than and smaller than FOV of 30°) and image dimensions.

Transformation to 30° disc-centered image: **CROPPING/EXTENSION**

Image	Processing step	Motivation
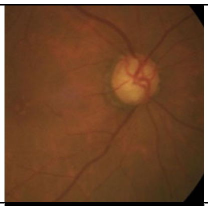	Original image	
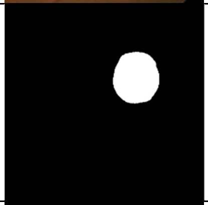	ONH localization	To compute the disc ratio for all individual images. This disc ratio can then be used to determine the FOV.
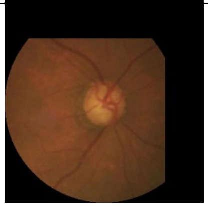	30° cropping with ONH centered	Disc ratio per image was divided by 0.23 (average disc ratio of 30° fundus image in G-RISK training data), crop factor ranged from 0.28 to 3.44
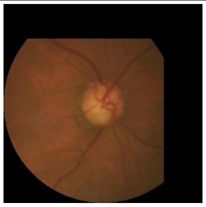	Resize image to 1444 x 1444	As there exists heterogeneity in image size, all images are uniformly resized to the dimensions of 30° images with which G-RISK was trained with
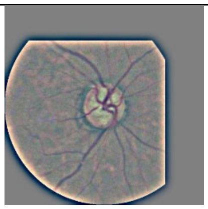	Lighting equalization	Lighting equalization, typically used in larger fundus images with larger FOV, was applied to approach the color distribution of G-RISK training images
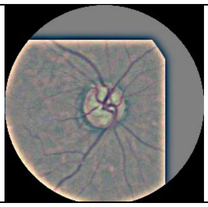	Circle crop with radius 1	To mimic the circular FOV encountered typically in 30° images, pixels outside the circle are replaced by a black color.

ODIR

ODIR fundus images show a lot of variety in FOV, and not all of the images contain a visible ONH.

Transformation to 30° disc-centered image: **CROPPING/EXTENSION**

Image	Processing step	Motivation
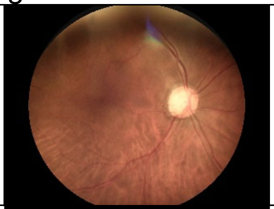	Original image	
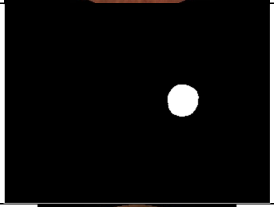	ONH localization	Required for cropping of 30° FOV image with centering on the ONH, in addition to the image quality control.
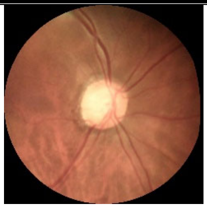	30° cropping with ONH centered	Disc ratio grouped by image dimensions was divided by 0.23 (average disc ratio of 30° fundus image in G-RISK training data), crop factors ranged from 0.33 to 1.13
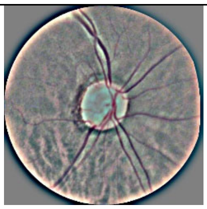	Lighting equalization	Lighting equalization, typically used in larger fundus images with larger FOV, was applied to approach the color distribution of G-RISK training images
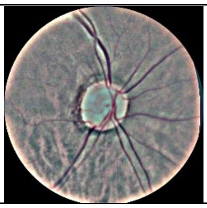	Circle crop with radius 1	To mimic the circular FOV encountered typically in 30° images, pixels outside the circle are replaced by a black color.

REFUGE2

REFUGE2 fundus images all feature a detected ONH, and were photographed at a 45° angle.

Transformation to 30° disc-centered image: **CROPPING**

Image	Processing step	Motivation
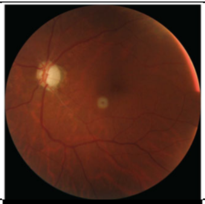	Original image	
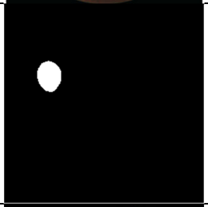	ONH localization	Required for cropping of 30° FOV image with centering on the ONH
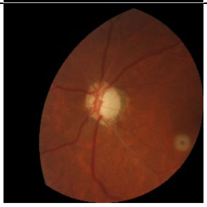	30° cropping with ONH centered	Disc ratio grouped by image dimensions was divided by 0.23 (average disc ratio of 30° fundus image in G-RISK training data). Crop factor was 0.69
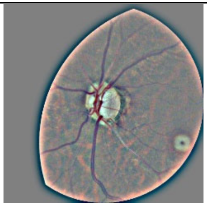	Lighting equalization	Lighting equalization, typically used in larger fundus images with larger FOV, was applied to approach the color distribution of G-RISK training images
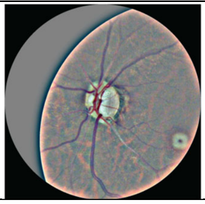	Circle crop with radius 1	To mimic the circular FOV encountered typically in 30° images, pixels outside the circle are replaced by a black color.

GAMMA

GAMMA fundus images all feature a detected ONH, and were photographed at a 45° angle.

Transformation to 30° disc-centered image: **CROPPING**

Image	Processing step	Motivation
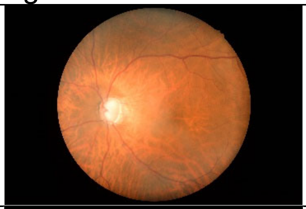	Original image	
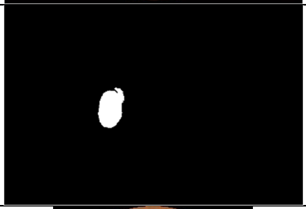	ONH localization	Required for cropping of 30° FOV image with centering on the ONH
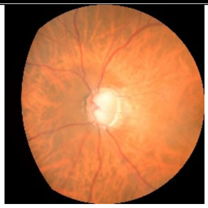	30° cropping with ONH centered	Disc ratio grouped by image dimensions was divided by 0.23 (average disc ratio of 30° fundus image in G-RISK training data). Crop factor = 0.69
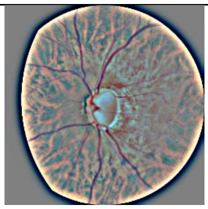	Lighting equalization	Lighting equalization, typically used in larger fundus images with larger FOV, was applied to approach the color distribution of G-RISK training images
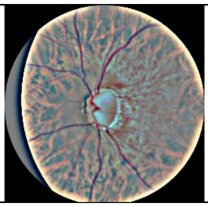	Circle crop with radius 1	To mimic the circular FOV encountered typically in 30° images, pixels outside the circle are replaced by a black color.

RIM-ONEr3

RIM-ONEr3 fundus images are all stereoscopically photographed, with horizontal FOV of 20° and vertical FOV of 27°.

Transformation to 30° disc-centered image: **EXTENSION**

Image	Processing step	Motivation
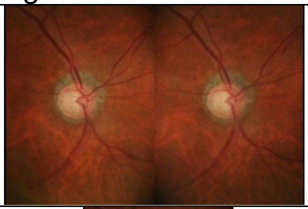	Original image	
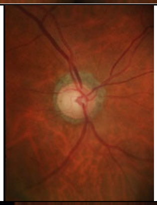	Stereoscopic image cropping	Cut off the right part of the stereoscopic image (preserve the left image)
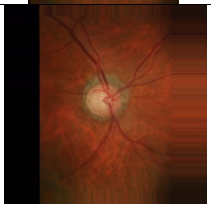	Border replicate padding	Border replicate padding was applied: 10% horizontally, 3° vertically. This corresponds to 268 pixels on horizontal sides, 79 pixels on vertical sides
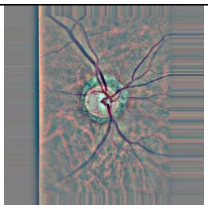	Lighting equalization	Lighting equalization, typically used in larger fundus images with larger FOV, was applied to approach the color distribution of G-RISK training images
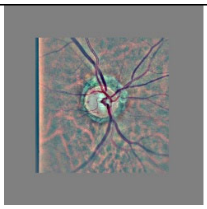	Square crop with radius 0.68	After lighting equalization, the fake image information should be removed. The pixels outside the original image are now filled with a gray color.
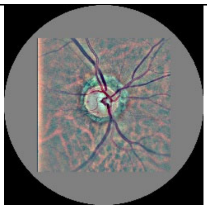	Circle crop with radius 1	To mimic the circular FOV encountered typically in 30° images, pixels outside the circle are replaced by a black color.

RIM-ONE DL

From the RIM-ONE DL data set paper: “all the images were cropped squarely around the head of the optic nerve using the same proportionality criterion”. This criterion was defined, hence an assumption of 28% of a 30° image was maintained.

Transformation to 30° disc-centered image: **EXTENSION**

Image	Processing step	Motivation
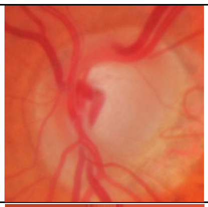	Original image	
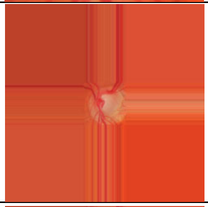	Border replicate padding	(imgdim / 28) * 100 = imgdim * 3.57 => Pad 1.79 x imgdim on each side
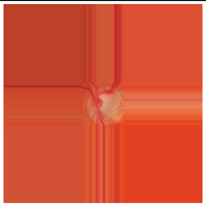	Resize image to 1444 x 1444	As there exists heterogeneity in image size, all images are uniformly resized to the dimensions of 30° images with which G-RISK was trained with
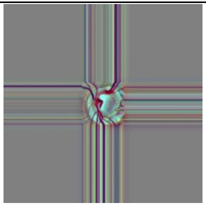	Lighting equalization	Lighting equalization, typically used in larger fundus images with larger FOV, was applied to approach the color distribution of G-RISK training images
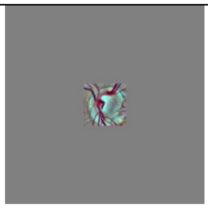	Square crop with radius 0.21	After lighting equalization, the fake image information should be removed. The pixels outside the original image are now filled with a gray color.
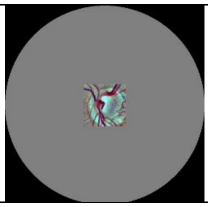	Circle crop with radius 1	To mimic the circular FOV encountered typically in 30° images, pixels outside the circle are replaced by a black color.

ACRIMA

ACRIMA fundus images are all systemically cropped around the ONH, with a bounding box of ONH size * 1.5, removing the unique ONH size information from the original fundus image.

Transformation to 30° disc-centered image: **EXTENSION**

Image	Processing step	Motivation
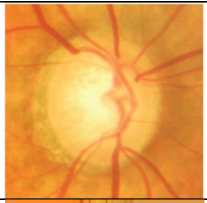	Original image	
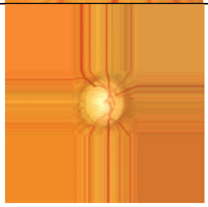	Border replicate padding	ACRIMA fundus images cover ~1/3 of a 30° fundus image. Therefore, border replicate padding at all sides was applied for scale correction
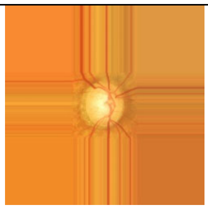	Resize image to 1444 x 1444	As there exists heterogeneity in image size, all images are uniformly resized to the dimensions of 30° images with which G-RISK was trained with
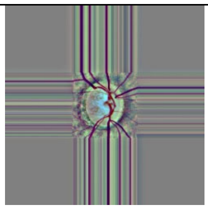	Lighting equalization	Lighting equalization, typically used in larger fundus images with larger FOV, was applied to approach the color distribution of G-RISK training images
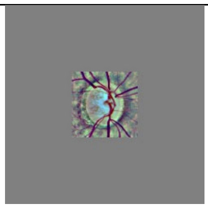	Square crop with radius 0.33	After lighting equalization, the fake image information should be removed. The pixels outside the original image are now filled with a gray color.
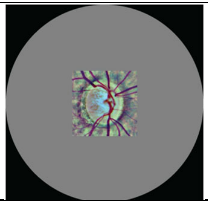	Circle crop with radius 1	To mimic the circular FOV encountered typically in 30° images, pixels outside the circle are replaced by a black color.

Supplementary Figure 3 – extreme FP and FN cases per data set, overlaid with saliency map

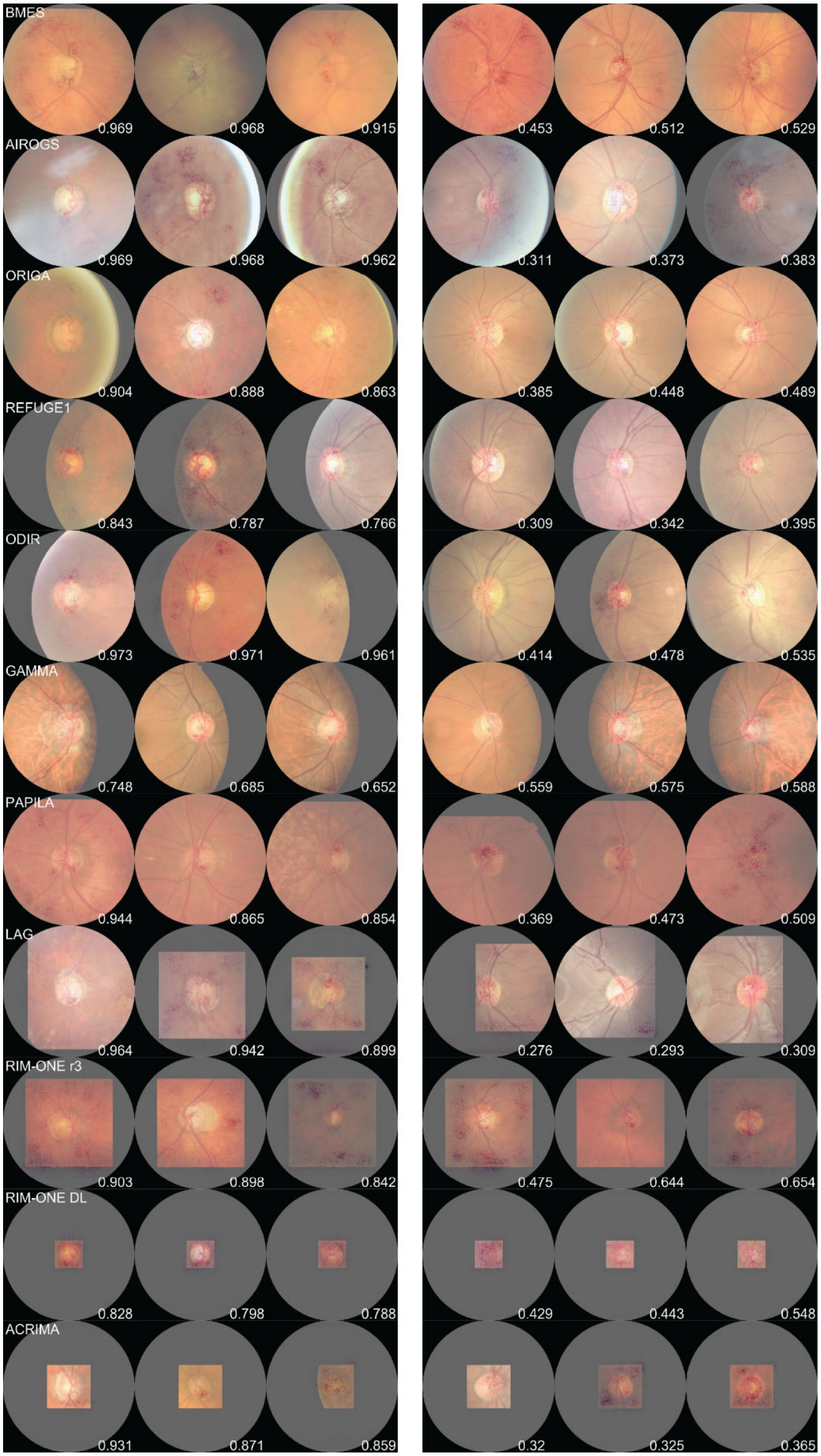

Supplement: Supplementary file 1 — Supplementary Material [file 41746_2023_857_MOESM1_ESM.pdf]
